# Promoting the co-utilisation of glucose and xylose in lignocellulosic ethanol fermentations using a data-driven feed-back controller

**DOI:** 10.1186/s13068-020-01829-2

**Published:** 2020-11-18

**Authors:** Pau Cabaneros Lopez, Isuru Abeykoon Udugama, Sune Tjalfe Thomsen, Christoph Bayer, Helena Junicke, Krist V. Gernaey

**Affiliations:** 1 Department of Chemical and Biochemical Engineering, PROSYS Research Center, Technical University of Denmark (DTU), Building 228A, 2800 Lyngby, Denmark; 2 Department of Geosciences and Natural Resource Management, University of Copenhagen, Frederiksberg C, Denmark; 3 Faculty of Process Engineering, Technische Hochschule Nürenberg, Nürenberg, Germany

## Abstract

**Background:**

The diauxic growth of *Saccharomyces cerevisiae* on glucose and xylose during cellulose-to-ethanol processes extends the duration of the fermentation and reduces productivity. Despite the remarkable advances in strain engineering, the co-consumption of glucose and xylose is still limited due to catabolite repression. This work addresses this challenge by developing a closed-loop controller that is capable of maintaining the glucose concentration at a steady set-point during fed-batch fermentation. The suggested controller uses a data-driven model to measure the concentration of glucose from ‘real-time’ spectroscopic data. The concentration of glucose is then automatically controlled using a control scheme that consists of a proportional, integral, differential (PID) algorithm and a supervisory layer that manipulates the feed-rates to the reactor accounting for the changing dynamics of fermentation.

**Results:**

The PID parameters and the supervisory layer were progressively improved throughout four fed-batch lignocellulosic-to-ethanol fermentations to attain a robust controller able of maintaining the glucose concentration at the pre-defined set-points. The results showed an increased co-consumption of glucose and xylose that resulted in volumetric productivities that are 20–33% higher than the reference batch processes. It was also observed that fermentations operated at a glucose concentration of 10 g/L were faster than those operated at 4 g/L, indicating that there is an optimal glucose concentration that maximises the overall productivity.

**Conclusions:**

Promoting the simultaneous consumption of glucose and xylose in *S. cerevisiae* is critical to increase the productivity of lignocellulosic ethanol processes, but also challenging due to the strong catabolite repression of glucose on the uptake of xylose. Operating the fermentation at low concentrations of glucose allows reducing the effects of the catabolite repression to promote the co-consumption of the two carbon sources. However, *S. cerevisiae* is very sensitive to changes in the glucose concentration and deviations from a set-point result in notable productivity losses. The controller structure developed and implemented in this work illustrates how combining data-driven measurements of the glucose concentration and a robust yet effective PID-based supervisory control allowed tight control of the concentration of glucose to adjust it to the metabolic requirements of the cell culture that can unlock tangible gains in productivities.

## Background

Cellulosic ethanol is commonly seen as a potential player to alleviate the dependence on fossil fuels. However, the low productivity of cellulose-to-ethanol fermentation limits its consolidation at an industrial scale. This low productivity is, among other reasons, associated with the diauxic growth on glucose and xylose [1, 2]. Even though new yeast strains able to utilise glucose and xylose simultaneously are currently being developed using genetic engineering, catabolite repression is still an issue, especially at high glucose concentrations [1, 2]. The simultaneous consumption of the two sugars can be stimulated from a process operation perspective, by keeping the concentration of glucose inside the fermenter below an inhibitory threshold, i.e. by running fed-batch fermentations. However, in cellulose-to-ethanol processes, advanced fed-batch control is challenging due to the substrate variability and to the weak correlation between the commonly monitored variables and the metabolic activity of the cell culture [3].

Fed-batch operations, where the substrate is fed continuously throughout the fermentation, is the most common operation mode in industrial biotechnology [4–9]. This is because fed-batch processes allow for a better utilisation of the carbon source (e.g., avoiding overflow metabolism [8, 9]) and limit the inhibition by substrate [8], resulting in increased productivities and higher titers of product [4] or biomass [5, 10]. By adjusting the substrate feed-rate during the fermentation, it is possible to change the concentration of substrates, products, or by-products inside the tank and to alter the metabolic activity of the fermentation hosts [7, 8]. Traditionally, the substrate feed is manually manipulated during the fermentation [5], or fixed to a constant flow rate. However, this strategy often leads to sub-optimal operations. Given the central role of substrate feed in the outcome of the fermentation, developing flow-rate control strategies is increasingly becoming an object of interest in industrial biotechnology [7] to respond to the market needs to increase production capacity [9]. The goal is then to find optimal feeding profiles that lead to specific control objectives (e.g., maximising the process productivity, or the biomass or product concentrations) [7, 8]. Open-loop controllers are often used to pre-define feeding profiles based on the known dynamics of the process. Kinetic models, coupled to mathematical programming routines, can be used to rigorously pre-define optimal feeding profiles accounting for the growth demands of the cell culture at the different stages of the fermentation [7, 11, 12]. While these strategies can result in improved operations, open-loop schemes are not able to reject process disturbances or to account for process variability because they lack ‘real-time’ process information, which limits their range of applicability. On the contrary, closed-loop controllers use ‘real-time’ process information to generate responses that meet the specific control objectives. The possibility to account for process disturbances and variability makes closed-loop schemes more robust than open-loop approaches. However, implementing closed-loop controllers in fermentation processes is challenging due to the non-linear nature of biological systems and the lack of ‘real-time’ process information (often limited to indirect measurements of cell growth such as pH, temperature or dissolved oxygen [10, 12]). Although feed-rate controllers have successfully been implemented using the commonly monitored variables [10–13], advanced measurements of substrate or products potentially result in tighter control of systems whose metabolism does not correlate well with the commonly monitored variables.

**Fig. 1:**
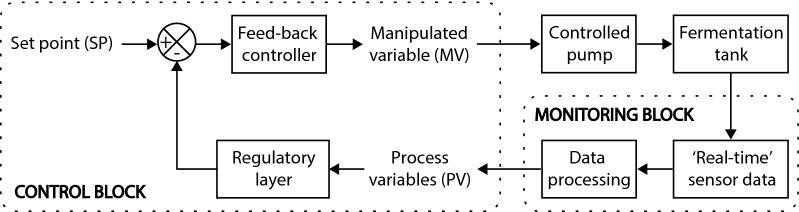
Structure of a closed-loop feed-back algorithm

In the present work, on-line spectroscopy was used to design and implement a closed-loop feed-back controller of the feed-rate in order to maintain the glucose concentration at a given set-point (SP), allowing to co-utilise glucose and xylose (Fig. 1). Attenuated total reflectance mid-infrared spectroscopy (ATR-MIR) was used to collect the spectra of the fermentation media continuously, and linear data-driven partial least squares (PLS) regression models were used to calculate the glucose concentration in ‘real-time’ from the collected spectra. Compared to other monitoring approaches to measure the concentration of glucose (such as at-line high-performance liquid chromatography or biosensors), ATR-MIR spectroscopy allows to automatically measure the concentration of glucose with high frequency directly from the fermentation media, thereby eliminating the need to prepare the sample [3]. Then, the predicted glucose concentration was used as the process variable (PV) for a proportional, integral, and differential (PID) algorithm that actuated to adjust the feed-rate of the reactor. A PID is one of the most common feed-back controllers used in industry. It attempts to control the error between a pre-defined SP and the actual value of the PV by generating a control signal [14] using the control law shown in Eq. 1.

1 $$u\left(t\right)=Kp\cdot e\left(t\right)+Ki{\int }_{0}^{t}e\left(\tau \right)d\tau +Kd\cdot \frac{d}{dt}\left(e\left(t\right)\right),$$

where $u(t)$ is the response signal, $e(t)$ is the error ($SP-PV(t)$), $Kp$, $Ki,$ and $Kd$ are the responses of the proportional, integral and differential terms of the controller and $\tau$ is the integration time ranging from 0 to $t$. A more detailed description of a PID controller can be found in specialised literature such as [14]. A supervisory layer based on PV manipulation concepts suggested in [15] was implemented to account for the non-linear behaviour of the fermentation kinetics and to avoid the undesirable scenarios where glucose accumulates above or is depleted below certain thresholds. The developed control scheme is a simple and effective approach to attain robust cellulose-to-ethanol fed-batch fermentation allowing tight control of the metabolic activity of the cell culture and stable operations within and between batches, even in the presence of substrate variability.

## Results

### Fermentation profiles and co-consumption of glucose and xylose

**Fig. 2:**
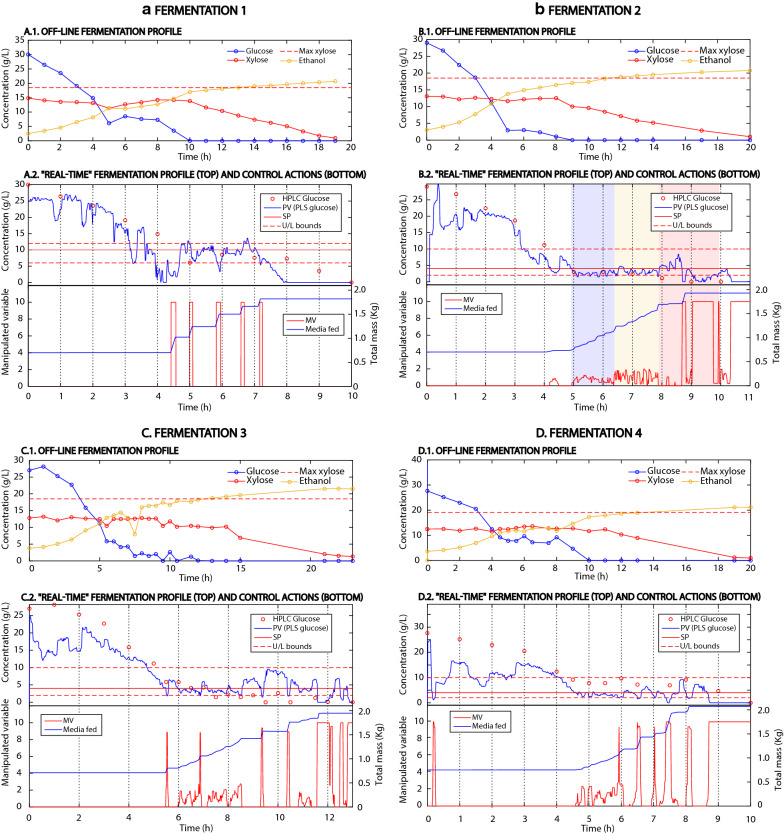
Experimental results of fed-batch fermentations 1–4. A.1–D-1 show the fermentation profiles of experiments 1–4. Note that initially, the 750 mL of biomass hydrolysate were mixed with 250 mL of inoculum, diluting the initial concentration of glucose and xylose. The dashed red line (Max xylose) shows the maximum xylose concentration accumulated in the reactor if there was no co-consumption of glucose and xylose. A.2–D-2 (top) show the PV (process variable) predicted by the PLS (partial least squares) model, the set-point (SP) for the fermentation, the upper and lower bounds, and the off-line samples analysed with high-performance liquid chromatography (HPLC). In fermentation 2 (B.2), the three different configurations of the PID that were tested are shown with blue, yellow, and red backgrounds. Note that before the first controller strategy was implemented in fermentation 2 (blue-shaded area) the same controller configuration as in fermentation 1 was used. A.2–D-2 (bottom) show the manipulated variable (MV), a signal scaled from 0–10 sent to the controlled pump, and the mass of wheat straw hydrolysate fed into the reactor

**Fig. 3:**
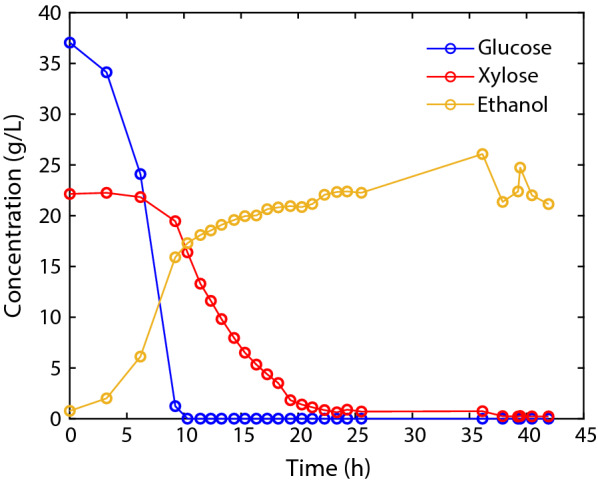
Profile of a batch fermentation (fermentation 5). The consumption of xylose only started when glucose was almost consumed entirely

The performance of four fed-batch fermentations (Fig. 2A.1–D.1) with feed-rate control was compared with a standard batch fermentation (Fig. 3), containing the same inoculum size (Table 1). Compared to the batch process, the fed-batch fermentations ran faster and had higher ethanol productivity (between 20 and 33% higher, Table 1), while exhibiting similar total ethanol yields on glucose and xylose (ethanol mass over the total glucose and xylose mass) as the batch process (~ 35%, Figs. 2A.1–D.1, 3). In order to account for the fact that different volumes were fermented throughout experiments 1–5, the ethanol production rate was calculated for the batch and fed-batch experiments, showing that in the fed-batch processes, ethanol was produced at a rate between 85 and 126% higher than in the batch process (Table 1). The co-consumption of glucose and xylose during the feeding phase was assessed by calculating the mass balance of xylose in the fed-batch experiments (Fig. 2A.1–D.1). In the scenario where yeast does not consume the two sugars simultaneously, xylose would accumulate in the reactor, reaching a maximum concentration of 19 g/L during the glucose-consumption phase. Note that the xylose concentration in the feed was higher than the initial concentration in the reactor because 750 mL of wheat straw hydrolysate were diluted with 250 mL of inoculum. However, during the fed-batch fermentations, xylose did not exceed 15 g/L (Fig. 2A.1–D.1), which indicates that operating the fermenter at a low glucose concentration promotes the co-consumption of glucose and xylose. Besides promoting the substrate co-consumption, operating at low glucose concentrations also reduces the concentration of the inhibitors inside the reactor, as glucose is only consumed after the inhibitors have been detoxified below an inhibitory threshold. This indicates that the increased productivity reached in the fed-batch fermentations, is not only the result of promoting the co-consumption of glucose and xylose, but also of controlling the inhibitory pressure inside the reactor (as some inhibitors such as furfural or 5-hydroxymethylfurfural are detoxified inside the reactor into less inhibitory compounds such as furfuryl alcohol [16]). Moreover, the pressure of acetic acid (another common inhibitor present in lignocellulosic hydrolysates) is controlled by maintaining the fermentation pH above the pKa of acetic acid, thus reducing the concentration of its protonated form and preventing it from diffusing into the cell [17]. The capability of *S. cerevisiae CEN.PK.*XXX to co-consume glucose and xylose during the fed-batch phase was studied by controlling the glucose concentration at 10 g/L (in fermentations 1 and 4) and 4 g/L (in fermentations 2–3). Note that while the set-point in fermentation 4 was 4 g/L of glucose, the bias in the PLS predictions resulted in an actual glucose concentration of 9–10 g/L (Fig. 2D.1). Whereas operating at a low glucose concentration clearly promoted the co-consumption of glucose and xylose, decreasing the set-point from 10 to 4 g/L did not improve the performance of the fed-batch fermentation (Table 1). This can be due to a reduction in the specific growth rate on glucose after lowering the concentration of glucose. When glucose and xylose are co-consumed, the total growth rate of the cell culture is the sum of the specific growth rate on each substrate. Reducing the concentration of glucose from 10 to 4 g/L increases the specific growth rate on xylose (due to lower catabolite repression), but also reduces the specific growth rate on glucose. These results indicate that there is an optimum concentration of glucose that maximises the overall cell growth. The results suggest that the benefits gained by reducing the effects of catabolite repression after lowering the glucose concentration from 10 to 4 g/L did not compensate for the subsequent decrease in the growth rate on glucose (Table 1). A risk associated with fed-batch operations in lignocellulosic-to-ethanol fermentation processes is the potential contamination by lactic acid bacteria (LAB). To detect potential contaminations by LAB, the concentration of lactic acid was measured every hour using HPLC. The HPLC analysis did not show lactic acid in any fermentations, indicating that LAB contamination did not occur in these particular fermentations runs carried out.

**Table 1:** Overview of fermentations 1–5

Fermentation	Mode	Initial volume (L)	Volume fermented (L)	Inoculum (g)	Time (h)	Ethanol productivity (g/L/h)	Ethanol production rate (g/h)
1	Fed-batch	1.00	1.81	1.75	19	1.13	1.94
2	Fed-batch	1.00	1.86	1.75	20	1.08	1.99
3	Fed-batch	1.00	1.86	1.75	22	1.02	1.90
4	Fed-batch	1.00	1.95	1.75	19	1.13	2.21
5	Batch	1.24	1.24	1.75	25	0.85	1.05

Productivities and production rates refer to ethanol formation. The fermented volume was calculated from the mass of media added in each fermentation, considering a density of 1.05 g/L. The ethanol productivity and production rate were calculated when the concentration of xylose dropped below 0.5 g/L

### Predictions of the glucose concentration using the PLS model

The PLS predictions of the glucose concentration in fermentations 1–4, were validated with off-line samples measured by HPLC (shown in Fig. 2A.2–D.2). The validation showed that the PLS performance drifted through fermentations 1–4. While in fermentations 1–3 it was able to accurately predict glucose concentrations below 25, 20, and 15 g/L, respectively, the PLS did not predict the glucose concentration accurately in fermentation 4 (Fig. 2 A.2-D.2). It was not possible to determine what caused the drift in the prediction quality of the PLS model throughout fermentations 1 to 4, however, the authors hypothesise that it might be caused by the inefficient cleansing of the flow-cell between fermentations, due to the impossibility to disassemble the apparatus. In fermentations 1–3, while the PLS model was not able to predict high glucose concentrations, it exhibited excellent performance predicting glucose below 15 g/L, so that the control objective in this study was met (i.e. to keep the glucose concentration at 10 g/L in fermentation 1 and at 4 g/L in fermentations 2–3). The error in predicting high glucose concentrations can be caused by the replacement of the ATR crystal and light source between the calibration and the application of the PLS models. Ideally, such a procedure would require a re-calibration of the PLS model; however, due to the limited availability of media and the excellent performance of the PLS models in the concentration range of interest during fermentation 1, this option was discarded. Calibrating PLS models to monitor fermentation processes is challenging due to the wide variety of compounds with highly overlapping spectra and due to the correlated dynamics of the different compounds [18–20]. In order to calibrate PLS models able to estimate glucose based on true correlations, larger calibration sets accounting for the variability of the different compounds and the fermentation matrix would be required [19–21]. The results showed that the calibration approach used in this work provides sufficiently robust PLS even after major changes were done to the instrument (Fig. 2A.2–D.2). This consistency in the PLS models was the result of decoupling the concentrations of glucose, xylose, and ethanol in the calibration set, which limited the interference of the fermentation dynamics with the PLS predictions. A limitation of the calibration set is the unaccounted effect of the change in the fermentation matrix, which can be significant and affect the PLS predictions. Therefore, even though the PLS models performed satisfactorily to meet the control objective, an extended calibration set, and a better description of the changes in the fermentation matrix would improve the robustness of the models.

### Performance of the controller

**Fig. 4:**
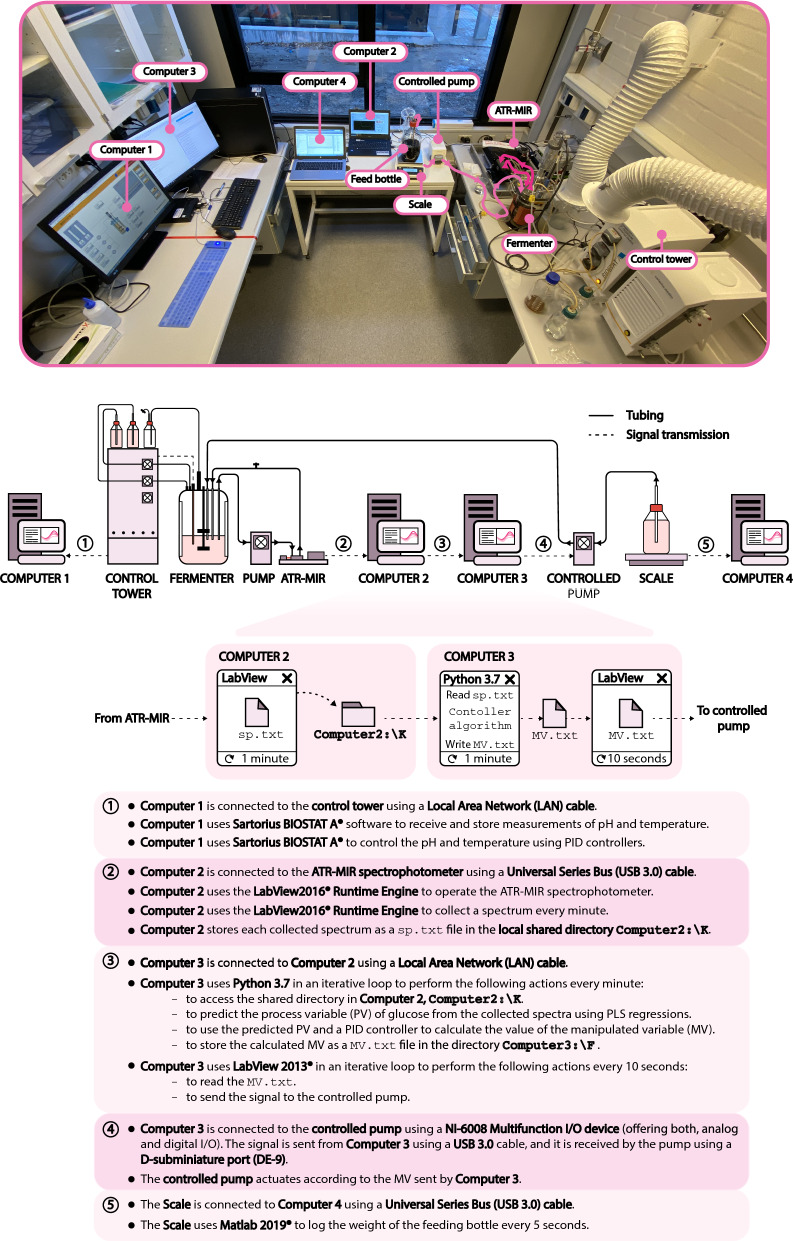
Experimental implementation of the controller. Note that four computers were required due to incompatibilities between the different softwares used in this process

The controller algorithm was tuned throughout fermentations 1–4 to achieve the necessary control response for maintaining the glucose concentration at the desired set-point (Table 3). To prevent the undesirable situations where glucose accumulates in the tank (limiting the co-consumption of glucose and xylose) or is consumed below a threshold (reducing the growth rate of the cell culture), an operational window around the SP was defined in the regulatory layer. When the glucose concentration was outside the upper and lower bounds (UB and LB, respectively), the PV was manipulated within the regulatory layer to increase the response of the controller (Table 3, Fig. 4, and Additional file 1). The non-linearity associated with the kinetics of the process were accounted for in the regulatory layer by manipulating the PID parameters (Table 3, Fig. 4, and Additional file 1). All the experiments were automatically operated by the different controllers and did not require any intervention. All fermentations began with a batch phase where yeast consumed glucose. When the glucose concentration dropped below the pre-defined SP, the fed-batch started, and the controller fed fresh media into the reactor until all the media was added. Then, the remaining xylose was consumed in a final batch phase (Fig. 2A.1–D.1). An overview of the performance of the different controllers is shown in Fig. 2A.2–D.2.

#### Fermentation 1: PID controller

The operational window for the first fermentation was defined between 12 and 8 g/L of glucose, respectively (Table 3). Since the dynamic response of the system was unknown before starting fermentation 1, an initial estimate for the PID tuning parameters was made based on the dynamics of a batch fermentation (fermentation 5, Table 3) using the lambda tuning method [22] and fundamental understanding of the process. Figure 2A.2 shows that the controller responded immediately to changes in the PV and actuated as an on/off controller alternating short feeding periods with longer non-feeding periods. The fed-batch phase lasted 3 h. The difference in length between feeding and non-feeding intervals revealed the different dynamics of the process. Although this configuration allowed for some control of the glucose concentration, the PV oscillated around the SP with a wide amplitude, and the controller let the PV exceed the pre-defined boundaries (Fig. 2A.2), creating an undesirable fluctuating environment that pushed the cell culture to change its metabolic activity continuously.

#### Fermentation 2: three PI controllers

In the second fermentation, the SP, UB, and LB were decreased to 4, 10, and 2 g/L, respectively, to study if operating at a lower glucose concentration would improve the co-consumption of glucose and xylose. Three different configurations of the controller algorithm were implemented to correct the deficiencies found in controller 1 (controllers 2.1, 2.2, and 2.3 in Table 3). The first controller implemented in the second fermentation (controller 2.1) had the following modifications:

- $Kp$ was decreased to 1/3 of the $Kp$ used in fermentation 1 to reduce the magnitude of the controller response.
- $Ki$ was manipulated within the regulatory layer to account for the different dynamics between feeding and non-feeding periods. $Ki$ was set to the average time of the feeding periods if PV was below SP, and to the average time of non-feeding periods if PV was above SP.
- $Kd$ was set to zero, turning the PID controller into a PI controller.

In contrast to the controller used in fermentation 1, controller 2.1 responded smoothly, and fed media continuously during the entire fed-batch phase, bringing the PV close to the SP with a small off-set error of approximately 1.5 g/L (Fig. 2B.2, blue-shaded area). However, the response was slow, and the controller let the PV drop below the LB on several occasions. An increase of Kp from 0.31 to 0.62 resulted in a reduction of the off-set error to 1 g/L and in a faster response when the PV dropped below the lower boundary (controller 2.2, Table 3). This situation is illustrated in Fig. 2B.2 (yellow shaded area). In order to eliminate the off-set error without compromising the stability of the response, the third controller (2.3) also manipulated $Kp$ within the regulatory layer (Table 3, Additional file 1). However, this strategy overshot the controller causing the PV to fluctuate around the SP (Fig. 2B.2, red-shaded area).

#### Fermentation 3: PI controller

In fermentation 3, the SP and operational window were defined as in fermentation 2. Increasing the $Kp$ from 0.62 to 0.72 (in controller 3, Table 3) resulted in a smooth response of the controller that successfully reduced the off-set error to 0.5 g/L during the first 3 h of the fed-batch phase. Also, when the PV fell below the lower boundary, the controller responded strongly to bring the glucose concentration back to the operational window (Fig. 2C.2). The dramatic drop in the glucose concentration, below the LB, at 8.5, 9, and 10.25 h of fermentation 3 caused a strong response of the controller resulting in the accumulation of glucose above the SP. These sudden changes in the PV, probably caused by noise in the PLS predictions, can compromise the stability of the controller, especially when the PV drops below the SP. Despite the noisy measurements, the controller was able to maintain the glucose concentration inside the operational window.

#### Fermentation 4: PI controller with rate regulator

Fermentation 4 was operated with the same SP, operational window, and controller configuration as fermentation 3 (Table 3). However, the rate of change in the PV between two successive iterations was limited to 2 g/L to increase the robustness of the controller towards noisy measurements (i.e. it was assumed that glucose could not change more than 2 g/L per minute, Fig. 4b). Although the PLS model did not predict the real glucose concentration accurately, the results of fermentation 4 still provide information about the performance of the controller. The rate regulator successfully improved the robustness of controller 4, which was able to keep the PV close to the SP and to re-conduct the PV back to the operational window without overshooting (Fig. 2C.2 and D.2). Even though the rate regulator was an efficient way to increase the robustness of the controller against noise in the measurements, other more advanced signal filters such as the Savitzky–Golay smoothing algorithm or extensions of the Kalman filter to non-linear systems would be a more suited approach.

## Discussion

The work presented in this manuscript shows how a new generation of “information rich” process measurements that are not the traditional temperature, flow and pressure measurements can add tangible value to fermentation processes when combined with targeted use of data-driven process monitoring methods and established closed-loop process control methods. To this end, this work differs from the traditional sensor/measurement development and process monitoring developments, which shows the potential benefits of a given monitoring strategy, but in general, fail to show tangible benefits by using the data monitored on-line to carryout closed-loop control [23–25]. Some recent studies have used glucose measurements taken using Raman spectroscopy [26] or total sugars content measured with the refractive index [27] together with P-controllers to adjust the feed flow-rate in ethanol fermentations. Although this resulted in faster fermentation processes, P-controllers often show off-set errors that prevent the present value from reaching the set-point and thus from the optimal conditions. Moreover, processes that only use the refractive index to monitor total sugar concentration are not able to measure the concentration of glucose alone, but only the total concentration of sugars instead. This can become a limitation, especially in processes that require operating at low glucose concentrations or in strains with a high sensitivity to changes around the set-point. The need for a fine control of the glucose concentration is illustrated in Table 1, where the drop in the glucose set-point from 10 g/L to 4 g/L caused a significant reduction in the process productivity. The PI controller implemented in the present work allowed for reducing the off-set error to match the glucose set-point. This allows tightening the fermentation operation to the metabolic requirements of the yeast strain to increase the productivity. In the present implementation, a single-input single-output (SISO) approach was used to effectively control the feed-rate of substrate during anaerobic fed-batch fermentations. However, if the PLS models were able to accurately monitor other state variables such as the xylose or ethanol concentrations, this opens up for the implementation of multiple-input single-output (MISO) or multiple-input multiple-output (MIMO) approaches, where several variables are considered to coordinate the response of the controller. *At the same time, even in a SISO configuration these state variables can be used to better manipulate the feed-rate taking into account effects such as non-linearities caused to the glucose consumption.* In lignocellulosic ethanol processes, it is common that the initial concentration of sugars differs between and within feedstocks, depending on the pretreatment. Ideally, higher sugar concentrations in the feed are desired to reduce the costs associated to the downstream operations. Higher substrate concentrations in the feed would result in longer fed-batch phases, which would extend the co-consumption of glucose and xylose.

This work also shows the value proposition of this targeted process automation, that is despite the relatively time-consuming control set-up and the need for a dedicated sensor and automated feed dosing capabilities, a 20–33% increase in productivity recorded justifies these efforts. To this end, this example stands as a proponent to value proposition for megatrends such as digitalisation and big data in the biomanufacturing industry.

## Conclusions

In the present work, a novel data-driven closed-loop feed-back controller of the feeding rate was implemented in order to promote the co-consumption of glucose and xylose during fed-batch cellulose-to-ethanol fermentations. The controller was based on on-line measurements of the glucose concentration inside the fermenter. This allowed for the automatic adjustment of the feed-rate to tightly meet the metabolic requirements of the cell culture, which promoted consuming glucose and xylose simultaneously, limiting the effect of the inhibitors, and increasing the fermentation productivity considerably. The closed-loop feed-back controller implemented in this work only needed four fed-batch fermentations to tune the PID parameters making it a practical and straightforward approach for ‘real-time’ based control of fed-batch fermentations. Since the controller responds to on-line measurements of the process variable, it can detect and respond in ‘real-time’ to disturbances in the process variable, and it is more robust to changes in the substrate composition than other control schemes such as open-loop approaches. The flexibility and robustness of the current experimental set-up allow for testing other advanced closed-loop controllers such as model-predictive approaches and pave the way towards the implementation of digital representations of the fermentation processes updated with ‘real-time’ data (digital twins).

## Materials and methods

### Cell culture propagation

One colony of the xylose-consuming *Saccharomyces cerevisiae* CEN.PK.XXX [28] strain was transferred from a YPX-agar plate (yeast extract 10 g/L (Microbiology Fermtech, Merck, New Jersey, USA), peptone from casein, 20 g/L (Microbiology Fermtech, Merck, New Jersey, USA) and xylose 20 g/L (Sigma Aldrich, Missouri, USA)), to a 250 mL shake flask containing 100 mL of liquid YPX media, and incubated at 30 ºC and 180 rpm. After 36 h of incubation, 2 mL of cell culture was transferred to two 500 mL shake flasks (1 mL each), containing 250 mL of YPX media and incubated at 30 ºC and 180 rpm for 36 h prior to inoculation.

### Fermentation experiments

Four fed-batch experiments (fermentations 1–4) and one batch (fermentation 5) experiment were conducted in a 2.5 L BIOSTAT® A bioreactor (Sartorius, Göttingen, Germany), equipped with two 6-bladed Rushton impellers, and pH, temperature and stirring speed control. The fermentation medium consisted of wheat straw hydrolysate, supplemented with 5 g/L of yeast extract and 10 g/L of peptone. The preparation and the composition of the wheat straw hydrolysate are described in the Additional file 1 and in Table 2, respectively. The fed-batch fermentations had an initial volume of 1 L to ensure the media covered the lower impeller. The feed-rate was adjusted automatically with the control algorithm actuating on a Watson-Marlow 114D peristaltic pump head (Watson Marlow, Falmouth, UK). Different control schemes were tested in fermentations 1–4 to understand the dynamics of the system and to tune the PID controller (Table 3) appropriately. The pH was kept at 6 using 5 M H_2_SO_4_ and 2 M NaOH, while the temperature and stirring rate were controlled at 30 ºC and 450 rpm, respectively. 250 mL of grown cell culture grown in YPX media (see Sect. 5.1) were inoculated to 750 mL of wheat straw hydrolysate media, resulting in an initial dry weight of ~ 2.5 g/L (measured as described in [29]). The fermentation was stopped when the xylose concentration dropped below 0.5 g/L. The amount of media supplied to the fermenter oscillated between 1.8 and 2.2 kg, depending on the fermentation. The batch fermentation had a constant volume of 1.4 L and the same inoculum size. On an hourly basis, 1.5 mL of fermentation media were withdrawn from the fermenter, filtrated through a 0.20 µm cellulose acetate filter (Labsolute, Renningen, Germany) and stored at – 20 °C until analysis with high-performance liquid chromatography (HPLC).

**Table 2:** Composition of the wheat straw hydrolysate

Compound	Concentration (g/L)
Glucose	39
Xylose	22
Furfural	0.62
5-HMF	0
Acetic acid	3.05

**Table 3:** Overview of the different control schemes. The set-points (SP), upper and lower boundaries (UB and LB, respectively), the parameters in the proportional ($Kp$), integral ($Ki$) and differential ($Kd$) control algorithm (PID) and the process variable (PV) conditioning according to the set-point are shown for each fermentation

Fermentation (controller)	Set-point and boundaries (g/L)			Regulatory layer											
	SP	UB	LB	If PV > SP				If PV = SP				If PV < SP			
				PV conditioning	PID parameters			PV conditioning	PID parameters			PV conditioning	PID parameters		
					$Kp$	$Ki$ (h^−1^)	$Kd$ (h)		$Kp$	$Ki$ (h^−1^)	$Kd$ (h)		$Kp$	$Ki$ (h^−1^)	$Kd$ (h)
1 (1)	10	12	8	PV = PV + SP	1.00	0.50	0.05	PV = PV	1.00	0.50	0.05	PV = PV − SP	1.00	0.50	0.05
2 (2.1)	4	10	2	PV = PV + SP	0.31	0.56	0	PV = PV	0.31	0.56	0	PV = PV − SP	0.31	0.11	0
2 (2.2)	4	10	2	PV = PV + SP	0.62	0.56	0	PV = PV	0.62	0.56	0	PV = PV − SP	0.62	0.11	0
2 (2.3)	4	10	2	PV = PV + SP	0.62	0.56	0	PV = PV	0.62	0.56	0	PV = PV − SP	0.80	0.11	0
3 (3)	4	10	2	PV = PV + SP	0.72	0.56	0	PV = PV	0.72	0.56	0	PV = PV—SP	0.72	0.11	0
4 (4)^a^	4^b^	10	2	PV = PV + SP	0.72	0.56	0	PV = PV	0.72	0.56	0	PV = PV − SP	0.72	0.11	0

^a^The regulatory layer in Fermentation 4, included a rate controller to prevent abrupt responses caused by noisy measurements

^b^The PLS model predicted the glucose concentration with a bias of 5 g/L, resulting in a real SP of 9 g/L and not 4 g/L

### Analysis with high-performance liquid chromatography (HPLC)

An UltiMate3000 HPLC (Thermo Scientific, Massachusetts, USA) loaded with an Aminex HPC-87 H column (BIORAD, California, USA) was used to measure the concentration of glucose, xylose, ethanol, acetic acid, and furfural off-line. The samples were derivatised by diluting 950 µL of the sample with 50 µL of 5 M H_2_SO_4_ and were run for 80 min at 50 °C with 5 mM H_2_SO_4_ as the mobile phase at a flow rate of 0.6 mL/min. All compounds were detected using the refractive index (RI) detector (ERC RefractoMax 520, Prague, Czech Republic).

### Spectroscopic analysis and calibration of the PLS regression model

The spectrum of the media was monitored on-line using an ATR-MIR spectrophotometer (NLIR APS, Farum, Denmark) which was provided by CellView IVS (Hillerød, Denmark) and equipped with a flow-cell connected to the fermenter using a closed recirculation loop. The residence time inside the closed recirculation loop was between 20–25 s with a flow-rate of 90 mL/min. Background and reference measurements were taken using air and having the laser turned off and on, respectively. The calibration samples were measured using an exposure time of 120 ms and 100 ATR spectra/min. The on-line measurements were performed using an exposure time of 85 ms and 80 averages. The different instrument settings used to calibrate the models and during the experiments were due to the replacement of the lamp and ATR crystal. The spectral range of the instrument was between 428 and 1833 cm^−1^ with a resolution of 1 cm^−1^. The models were built in Python 3.7 using the MBPLS [30]. The calibration set consisted of 21 semi-synthetic samples thoroughly designed following a design of experiments approach to minimise any correlation between the concentrations of glucose, xylose, and ethanol, which were considered the major contributors to the covariance of the spectral matrix [18, 19]. The pairwise Pearson correlation coefficient of glucose, xylose, and ethanol of 100,000 randomly generated Latin hypercubes [31] were calculated, and the design with a lower averaged correlation was selected. Real fermentation samples were not included within the calibration set to ensure that process dynamics did not interfere with the PLS predictions. A ‘static’ fermentation matrix (fermentation media without glucose, xylose, and ethanol taken at the end of the fermentation) was obtained by first fermenting 1 L of wheat straw hydrolysate (as described in “Materials and methods”, “Fermentation experiments”) and then stripping out the ethanol using sterile air for 24 h, and was used to create the semi-synthetic calibration set. The experimental space used to calibrate the PLS models was 0–40 g/L for glucose, 0–25 g/L for xylose and 0–22 g/L for ethanol. A PLS1 model with 4 latent variables was selected by minimising the root-mean square error during a leave-one-out cross validation routine (the RMSECV was equal to 1.45 g/L).

### The experimental set-up, the flow of data and the controller algorithm

The physical implementation of the controller, including the required hardware, the communication schemes between the different components, and the flow of data throughout the system, is explained in detail in Fig. 4.

**Fig. 5:**
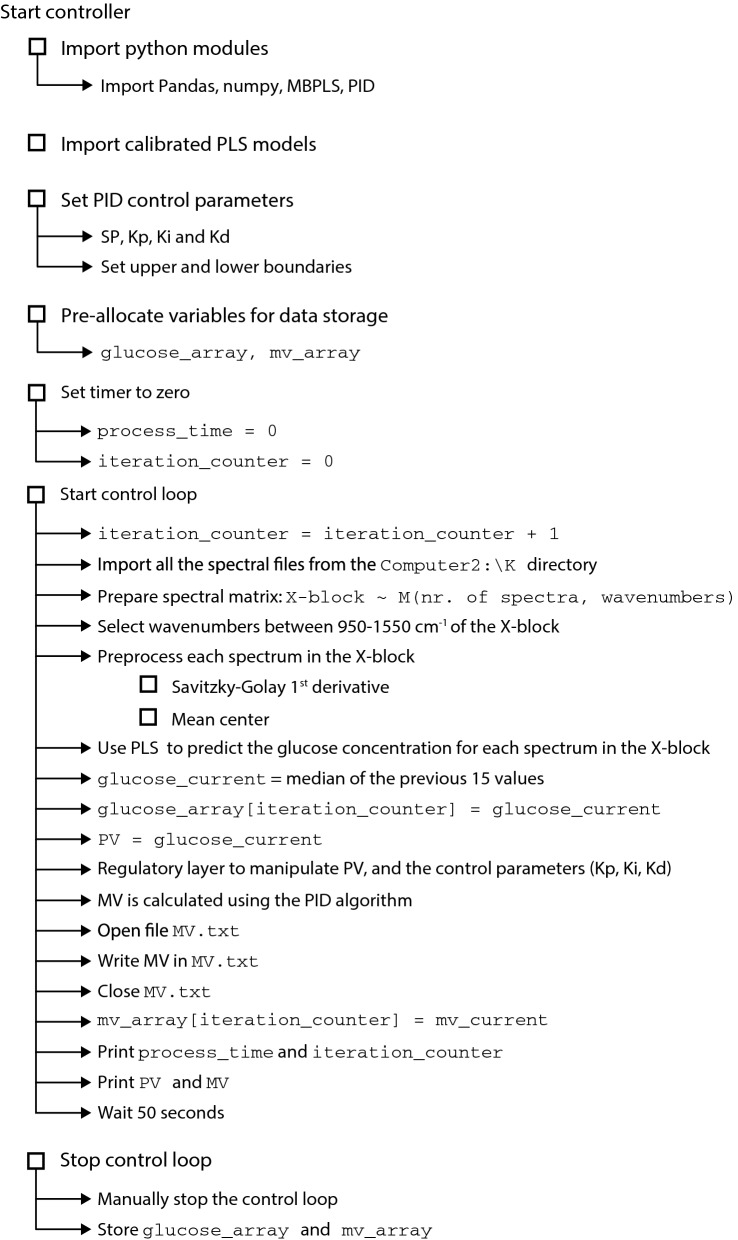
Structure of the controller algorithm. Note that different regulatory layers were used in fed-batch fermentations 1–4

The ATR-MIR spectrophotometer, Computers 2 and 3, and the controlled pump comprise the core components for feed-rate control, while Computers 1 and 4 are used to operate the fermenter and to record the weight of media fed into the reactor, respectively (Fig. 4). Every minute, the ATR-MIR spectrophotometer [operated from Computer 2 using LabView2016® Runtime Engine (National Instruments, Texas, USA)] stores a new spectrum as an ‘sp.txt’ file in a local folder shared with Computer 3. Then, the controller algorithm, written in Python 3.7 and implemented in Spyder 4.0.0 (https://www.spyder-ide.org/), runs iteratively every minute in Computer 3 to read the spectral data stored in Computer 2 and to generate a control output. The controller algorithm is shown in Fig. 5.

**Fig. 6:**
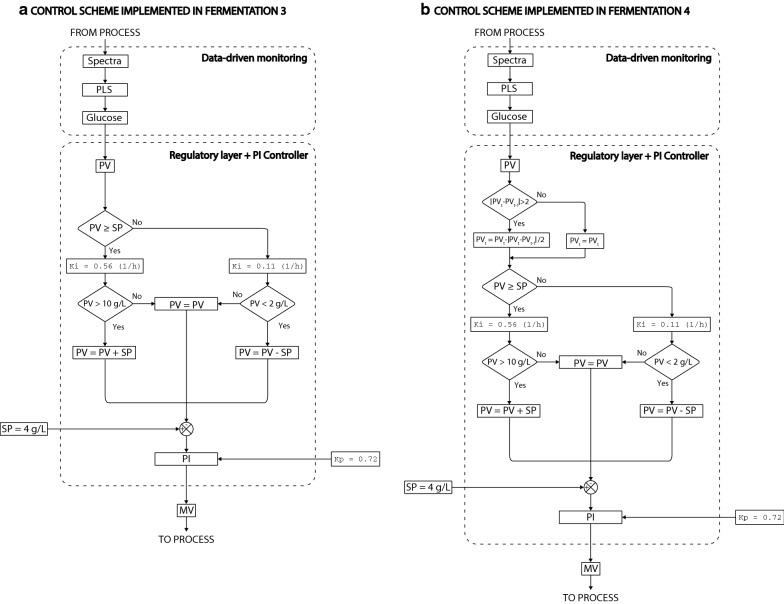
Control schemes of the regulatory layers used in fed-batch fermentations 3 and 4. Firstly, a partial least squares model (PLS) is used to calculate the process variable (PV) of the glucose concentration. Secondly, the PV is manipulated depending on the pre-defined set-point (SP) and the upper and lower boundaries. The integral term ($Ki$) of the proportional, integral (PI) controller is also manipulated depending on the PV, while the proportional term ($Kp$) is kept constant. The manipulated variable (MV), resulting from the PI controller, is sent to the actuator (controlled pump). The control schemes for the fed-batch fermentations 1 and 2 are shown in Additional file 1

The system is initialised by importing the calibrated PLS models, and by defining the set-point, the upper and lower boundaries and the control parameters. Then, the control loop starts and sequentially imports the spectra from the shared folder in Computer 2. The computer 3 then calculates the PV (i.e. the glucose concentration) using the calibrated PLS model and inputs this information to the supervisory control layer. The supervisory control layer then conditions the PV according to the regulatory layer and passes it to the PID controller (using the python library simple-PID) for the generation of the MV (output) in the form of an ‘MV.txt’ file. These actions happen in “real time” during batch operations. The supervisory layer is coded using if-logic and allows to define the operational window and to manipulate both, the PV and the PID controller parameters ($Kp$, $Ki$, and $Kd$), in each iteration depending on the measured PV and the upper and lower boundaries. This strategy helps to account for the different dynamics occurring when the PV is below or above the SP. Different configurations of the supervisory layer and tuning parameters for the PID algorithm were tested in fermentations 1–4 to optimise the performance of the controller (an overview of the different controllers is shown in Table 2, the control schemes for fermentations 3 and 4 are shown in Fig. 6, and the remaining control schemes are shown in Additional file 1.

The output of the controller algorithm was an MV signal scaled between 0 and 10, which corresponded to a flow-rate between 0 and 31.05 mL/min. The linearity between the MV and the flow-rate was assessed experimentally with four replicates to account for possible non-linearities in the pump’s behaviour (Additional file 1). The output MV (stored as an ‘MV.txt’ file) is accessed every 10 s by LabView2013® (National Instruments, Texas, USA) and forwarded first to the NI-6008 Multifunction I/O Device (National Instruments, Texas, USA) and then to the controlled pump. In order to prevent errors in accessing simultaneously the shared ‘MV.txt’ file (it is accessed by the controller algorithm and by LabView2013®), the file is only opened right before writing or reading the MV signal and closed immediately after. On a technical note, to avoid slow response times caused by phase delay between timers, LabView2013® iterates at a faster rate (every 10 s) than the controller algorithm (every minute), ensuring a fast response of the system.

## Supplementary information

**Additional file 1.** Supplementary material.

## Data Availability

The datasets used and/or analysed during the current study are available from the corresponding author on reasonable request.
