# Supplementary material for: Promoting the co-utilisation of glucose and xylose in lignocellulosic ethanol fermentations using a data-driven feed-back controller
Source: Biotechnol Biofuels. 2020 Nov 18;13:190. doi: 10.1186/s13068-020-01829-2 (PMC7672843; doi:10.1186/s13068-020-01829-2)
Supplement: Supplementary file 1 — Additional file 1. Supplementary material. [file 13068_2020_1829_MOESM1_ESM.docx]

**Supplementary material**

1. **Media preparation**
   1. **Pretreatment**

Pilot-scale steam explosion pretreatment was carried out at Lund University in the Department of Chemical Engineering (Sweden) on rolled dried wheat straw (WS) provided by TK Energy ApS (Denmark). 10 kg of WS was soaked in 90 kg of water for an hour (mass ratio 1:10). The soaked WS was press filtered at 200 bar for approximately 3-5 minutes until no more water was coming out of the unit (45 % DM). The pretreatment was performed in a steam pretreatment unit at 200 °C, and 16.8 bar on 1.5 kg of moist washed WS. The pretreatments were stopped with rapid pressure release after 10 min, where the material was flushed into a collection tank (steam explosion pretreatment). The pretreatment was done 14 times (9.5 kg DM), yielding 41.4 kg of pretreated material at 20 % DM (8.4 kg DM). The pretreated WS was stored at 4 °C until use.

- 1. **Enzymatic saccharification**

Enzymatic saccharification on the pretreated WS was performed at Lund University in the Department of Chemical Engineering (Lund, Sweden) using a 150 L reactor filled half. Hydrolysis was performed at 50 °C for 72 h, with stirring at 300 rpm. The enzyme dosage was 5 ml Cellic CTec2 (Novozymes A/S, Bagsværd, Denmark) per kg of slurry. The pretreated material was diluted to 10 % to enable stirring and was adjusted to pH 4.8 with 50 % NaOH. After hydrolysis, the material was centrifuged at 4000 rpm for 20 minutes (Heraeuse Multifuge X3R, Thermo Scientific, Massachusetts, USA) to reduce the solid contents, divided into containers of 5 liters, and stored at -20 °C until use.

1. **Control schemes used in fermentations 1 and 2**


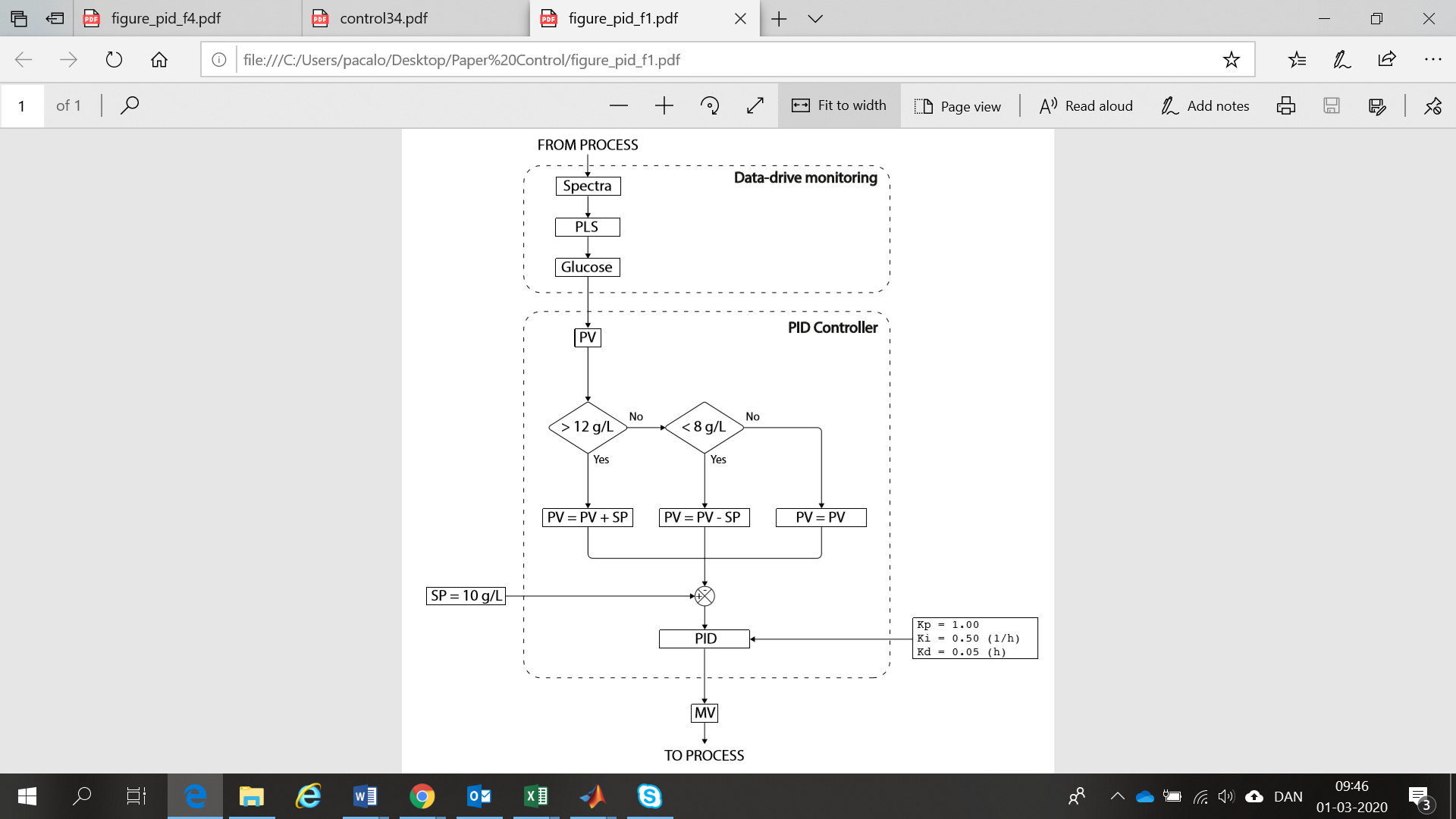


**Figure A.1.** The control scheme of the regulatory layer used in fed-batch fermentation 1. Firstly, a partial least squares model (PLS) is used to calculate the process variable (PV) of the glucose concentration. Secondly, the PV is manipulated depending on the pre-defined set-point (SP) and the upper and lower boundaries. The manipulated variable (MV), resulting from the PI controller, is sent to the actuator (controlled pump).


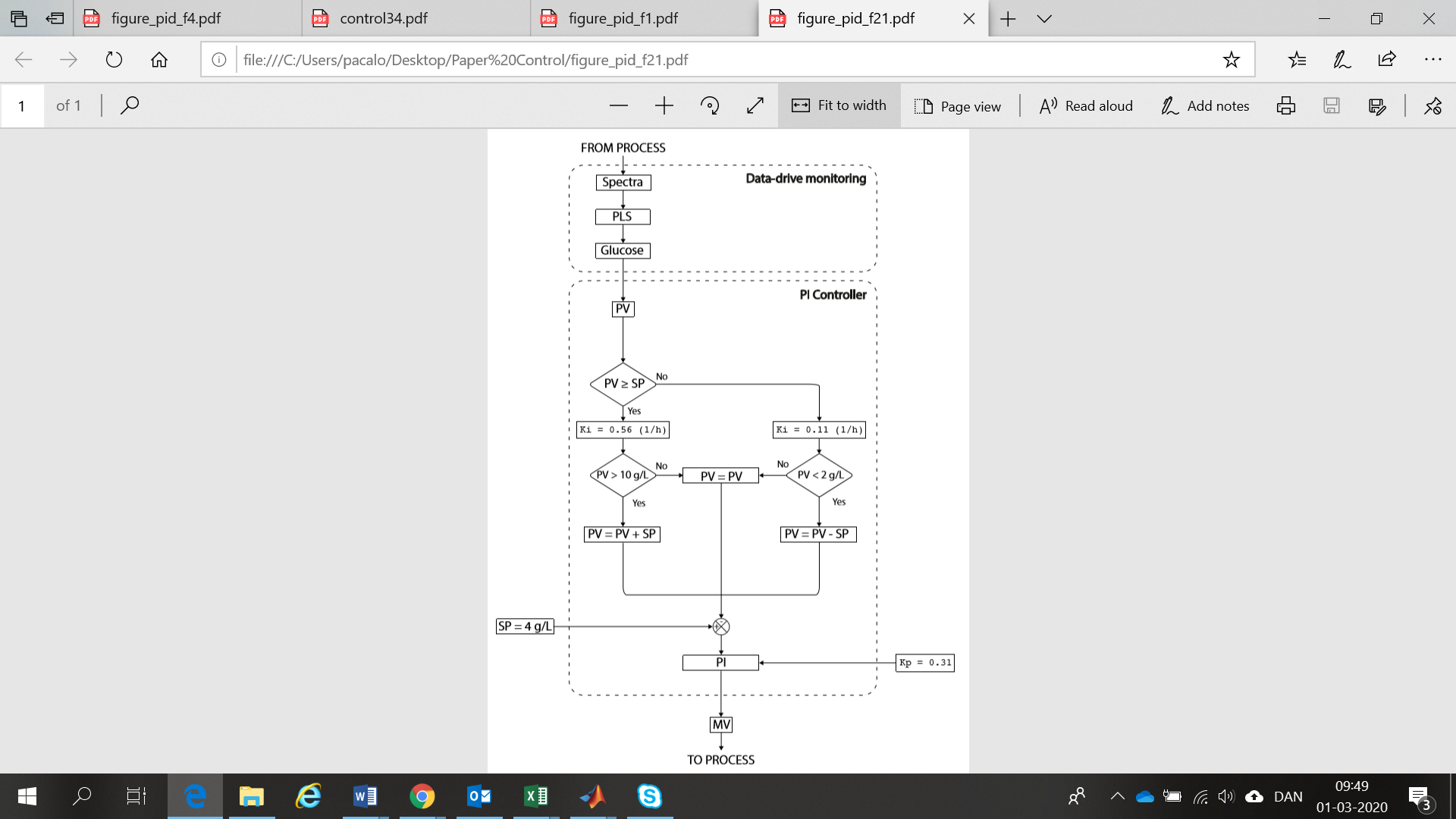


**Figure A.2.** The control scheme of the regulatory layer used in fed-batch fermentation 2.1. Firstly, a partial least squares model (PLS) is used to calculate the process variable (PV) of the glucose concentration. Secondly, the PV is manipulated depending on the pre-defined set-point (SP) and the upper and lower boundaries. The integral term ($Ki$) of the proportional, integral (PI) controller is also manipulated depending on the PV, while the proportional term ($Kp$) is kept constant. The manipulated variable (MV), resulting from the PI controller, is sent to the actuator (controlled pump).


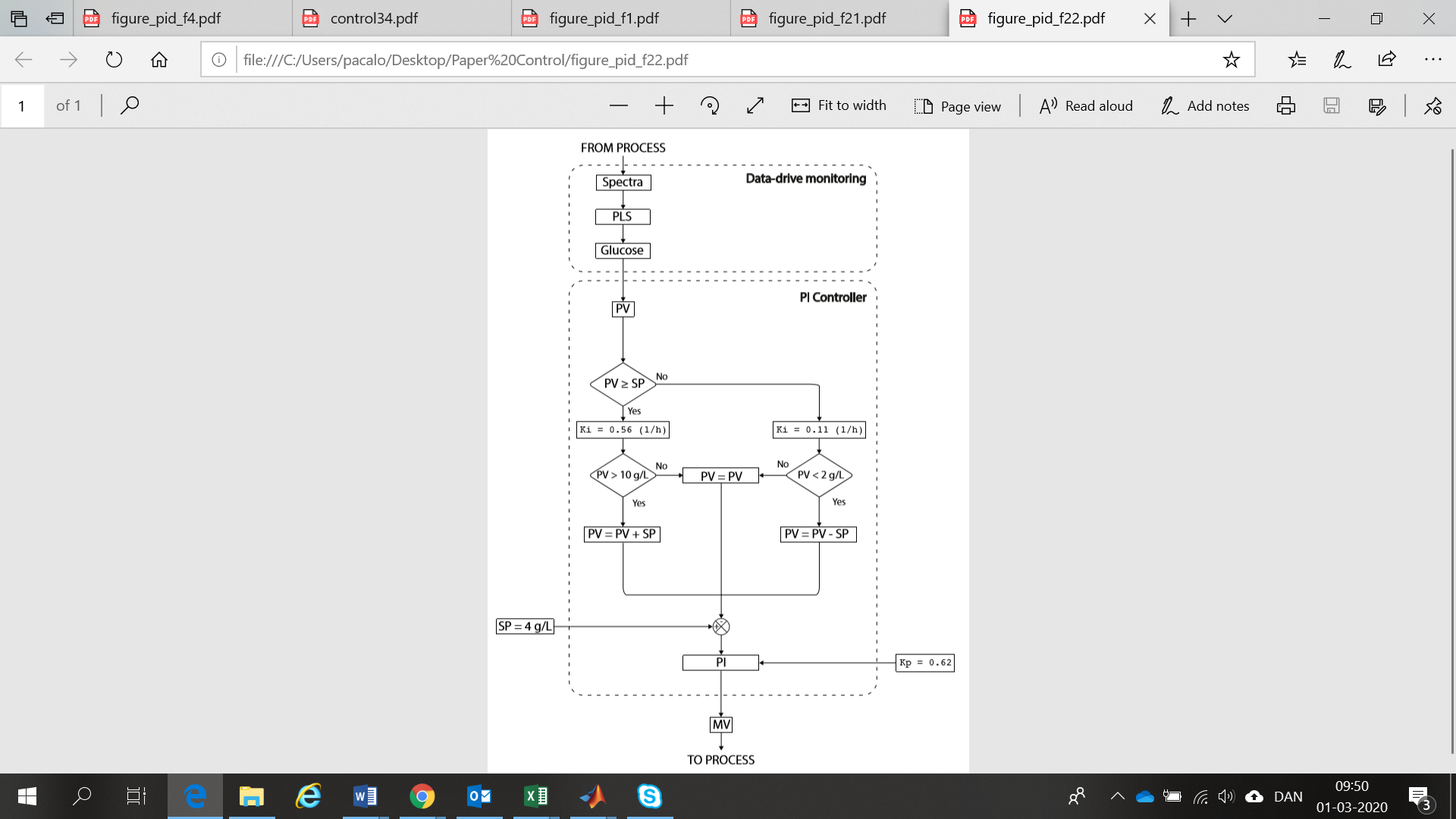


**Figure A.3.** The control scheme of the regulatory layer used in fed-batch fermentation 2.2. First, a partial least squares model (PLS) is used to calculate the process variable (PV) of the glucose concentration. Secondly, the PV is manipulated depending on the pre-defined set-point (SP) and the upper and lower boundaries. The integral term ($Ki$) of the proportional, integral (PI) controller is also manipulated depending on the PV, while the proportional term ($Kp$) is kept constant. The manipulated variable (MV), resulting from the PI controller, is sent to the actuator (controlled pump).


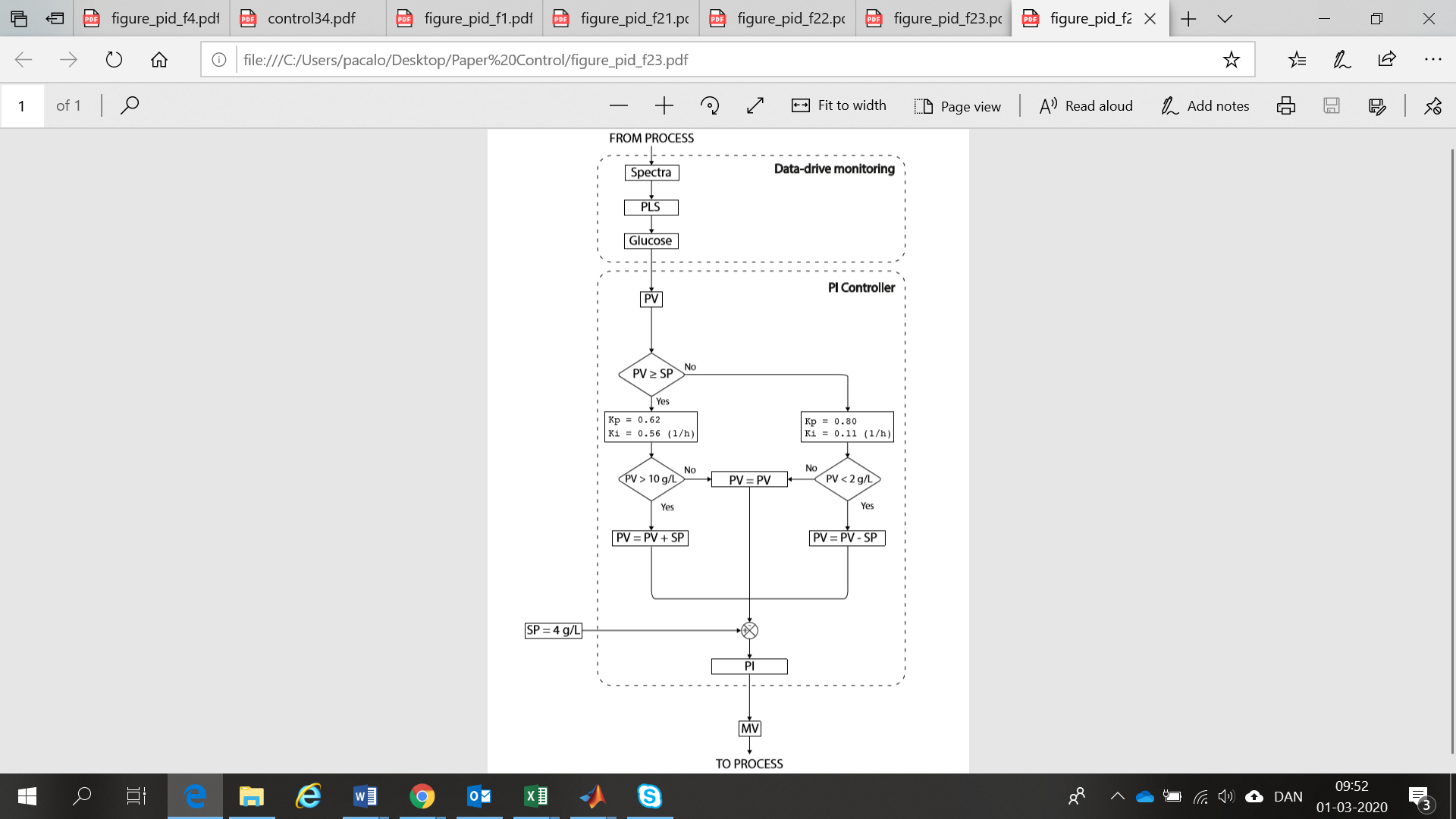


**Figure A.4.** The control scheme of the regulatory layer used in fed-batch fermentation 2.3. Firstly, a partial least squares model (PLS) is used to calculate the process variable (PV) of the glucose concentration. Secondly, the PV is manipulated depending on the pre-defined set-point (SP) and the upper and lower boundaries. Both, the proportional and the integral terms ($Kp$ and $Ki$ respectively) of the proportional, integral (PI) controller, are also manipulated depending on the PV. The manipulated variable (MV), resulting from the PI controller, is sent to the actuator (controlled pump).

1. **Calibration of the controlled pump**

The controlled pump was calibrated by correlating the set-point (arbitrary scale from 0 to 10) with its volumetric flow-rate. The mass of water displaced by the pump in one minute was measured using a scale (Sartorius, Göttingen, Germany). Four replicates were done, and the mean and standard deviation are shown in **Figure A.5.** It was assumed that the density of deionised water at 22 °C is 1 g/mL.


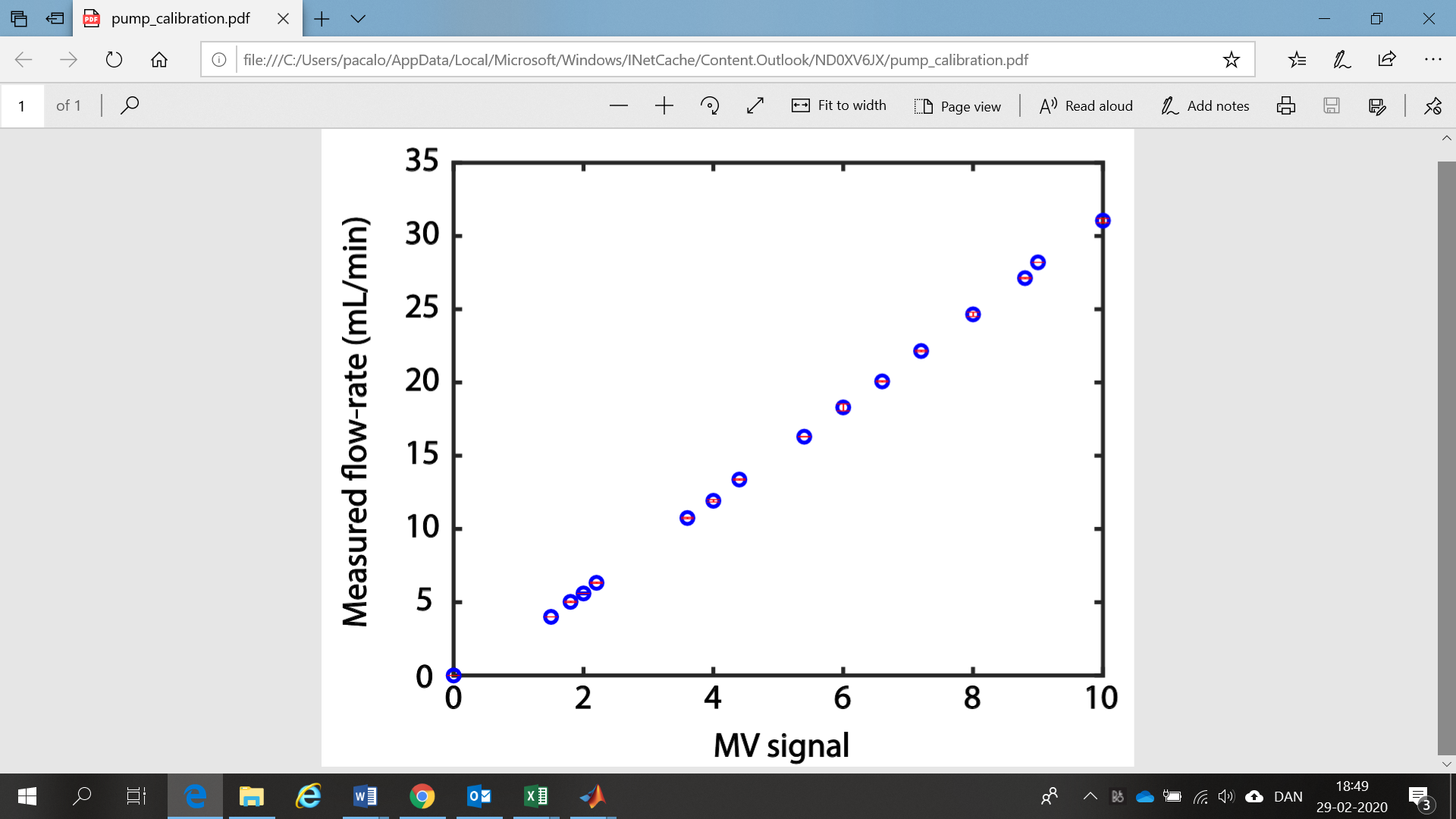


**Figure A.5.** Linearity between the scale of the signal sent to the pump by the controller and the volumetric flow-rate of the pump. A density of 1 g/mL was assumed. MV stands for the manipulated variable, and it is the signal sent by the PID controller.
